# Crystal Structures of Cyanine Fluorophores Stacked onto the End of Double-Stranded RNA

**DOI:** 10.1016/j.bpj.2017.10.002

**Published:** 2017-12-05

**Authors:** Yijin Liu, David M.J. Lilley

**Affiliations:** 1 Cancer Research UK Nucleic Acid Structure Research Group, MSI/WTB Complex, The University of Dundee, Dundee, United Kingdom

## Abstract

The indodicarbocyanine fluorophores Cy3 and Cy5 are extensively used as donor-acceptor pairs in fluorescence resonance energy transfer experiments, especially those involving single molecules. When terminally attached to double-stranded nucleic acids via the 5′ phosphate group these fluorophores stack onto the ends of the molecule. Knowledge of the positions of the fluorophores is critical to the interpretation of fluorescence resonance energy transfer data. The positions have been demonstrated for double-stranded (ds) DNA using NMR spectroscopy. Here, we have used x-ray crystallography to analyze the location of Cy3 and Cy5 on dsRNA, using complexes of an RNA stem-loop bound to L5 protein determined at 2.4 Å resolution. This confirms the tendency of both fluorophores to stack on the free end of RNA, with the long axis of the fluorophores approximately parallel to that of the terminal basepair. However, the manner of interaction of both Cy3 and Cy5 with the terminus of the dsRNA is significantly different from that deduced for dsDNA using NMR. The fluorophores are stacked on the terminal basepair such that their indole nitrogen atoms lie on the major groove side, and thus their pendant methyl groups are on the minor groove side.

## Introduction

Fluorescence resonance energy transfer (FRET) has been extensively used in single-molecule studies of nucleic acid structure and dynamics (1, 2, 3). Most commonly, this provides information on how distances between two fluorophores within DNA, RNA, or their complexes with proteins alter during structural transitions. The basis of this is the change in the efficiency of FRET (*E*_FRET_) as the interfluorophore distance changes. Transfer of electronic excitation energy is due to coupling between the donor emission dipole vector and the excitation dipole vector of the acceptor. The scalar product of these vectors is a function of the angle between them, and this complicates the extraction of absolute distance information from FRET data (4, 5, 6). If both fluorophores are mobile the analysis is simplified (5), but this is not always the case. In such circumstances, the interpretation will be aided by a knowledge of how the fluorophores are oriented relative to the macromolecule to be studied.

**Figure 1:**
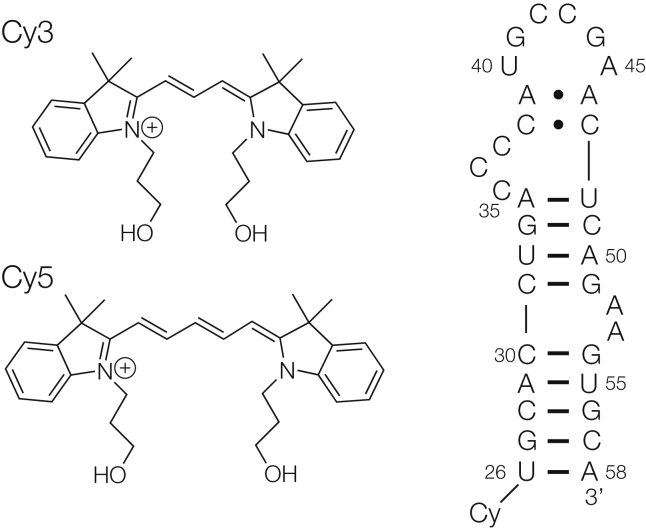
The chemical structures of Cy3 and Cy5 (*left*), and the sequence of the 33 nt stem-loop derived from 5S rRNA (*right*). Four forms of the RNA have been studied, with the four possible terminal basepairs. The fluorophores are connected to the 5′-terminus of the RNA (Cy) via one of the C_3_ linkers attached to the indole nitrogen atom.

In single-molecule FRET experiments it is helpful if the donor and acceptor fluorophores are chemically and spectroscopically similar. They should also be bright and photostable. One of the most commonly employed FRET pairs comprises the indodicarbocyanine fluorophores Cy3 and Cy5 (7). These are a subclass of the polymethine group of fluorophores (8), where a C_3_ or C_5_ polymethine linker connects two indolenine ring systems (Fig. 1). Polymethylene chains are attached to the ring nitrogen atoms, one of which is used to tether the fluorophore to the macromolecule, most frequently via the 5′-terminus of DNA or RNA. Cy3 and Cy5 have very high absorption cross sections (extinction coefficients of 135,000 and 250,000 L mol^−1^ cm^−1^, respectively) and quantum yields of 0.15 and 0.3, respectively. They are also reasonably photostable, especially in the presence of oxygen-scavenging systems and triplet state quenching. Although the spectroscopic properties are favorable, when terminally attached to double-stranded nucleic acids they are at least partially oriented. Therefore, the transition moment orientation factor κ^2^ cannot be assumed to be a constant, such as taking the value of 2/3, which would be appropriate for fluorophores that undergo rapid reorientation during the lifetime of the excited state (5). However, if the position of the fluorophore is known, then this can be an advantage in the interpretation of FRET data. Thus, establishing the location of the fluorophores is critical in the interpretation of such data, and consequently in the determination of the structure of the nucleic acid.

In experiments where Cy3 terminally attached to double-stranded (ds) DNA via a C_3_ linker was used as an energy transfer acceptor from fluorescein, we concluded that it was close to the helical axis. We reasoned that it could stack on the end basepair of the DNA helix, and demonstrated that this was indeed the case using two-dimensional NMR spectroscopy (9). We subsequently showed that Cy5 terminally attached to dsDNA was similarly stacked on the end of the helix (10). In both cases, the cyanine fluorophores took up positions akin to an additional basepair, with the distal indole ring stacked over the 3′ nucleobase. Terminal stacking is consistent with relatively high fluorescence anisotropy values of ≥0.3. The exact position adopted by the fluorophores was influenced by the nature of the attachment to the DNA, and we found that the rotational setting of cyanine fluorophores altered when tethered by a longer linker (11, 12).

These results showed that the cyanine fluorophores are constrained on the ends of dsDNA helices and that, consequently, orientation effects in energy transfer should be significant. This was demonstrated by studying FRET between Cy3 and Cy5 attached to the 5′-termini of dsDNA duplexes of systematically varied length. If both fluorophores are stacked onto the terminal basepairs then κ^2^ would be expected to vary sinusoidally with helix length, with two maxima and two minima per helical turn. This was performed for dsDNA of 10–24 bp, and the results were in excellent agreement with that predicted for a B-form helix (10). The experiment was repeated for a DNA-RNA helix, whereupon the phase changed to one consistent with an A-form helix. Although all these data clearly demonstrate a strong tendency for the cyanine fluorophores to stack on the terminal basepairs of double-stranded nucleic acids, simulation of the profiles of *E*_FRET_ with helix length indicated that the fluorophores were not rigidly fixed, but rather able to undergo in-plane lateral motion of significant amplitude (10). Additionally, fluorescent lifetime measurements showed that a fraction of the fluorophore at a given time (e.g., 0.17 for Cy3 attached to dsDNA) was unstacked from the helix (10, 13, 14).

In principle, FRET experiments on nucleic acids using cyanine fluorophores can provide valuable orientational information over and above estimates of distance, but since all information obtained is relative to the position and orientation of the fluorophores themselves, it is important to know how these are fixed in relation to the nucleic acid. For dsDNA NMR spectroscopy has provided this conformational information, but there are no similar data for RNA. However, some of the most interesting problems that could be addressed with this methodology are probably in RNA (15, 16), and so we want to learn how the cyanine fluorophores interact with RNA helices, and whether this differs from the interaction with dsDNA.

In this study, we have taken an x-ray crystallographic approach to study the conformation of Cy3 and Cy5 fluorophores attached to the 5′-termini of dsRNA molecules. Conscious of potential problems posed by interaction with neighboring molecules in the lattice, we sought an RNA-protein complex that should leave the free end of an RNA duplex devoid of macromolecular contacts. After considering a number of possible candidates, we opted to study a complex of a 34 nt stem-loop derived from 5S rRNA with the L5 protein of *Thermus thermophilus* that was previously solved by Perederina et al. (17). The protein principally interacts with the terminal loop of the RNA, whereas the free end of the RNA is flush-ended (i.e., perfectly base-paired), and projects away from the protein. We have obtained crystals of the protein bound to the stem-loop RNA, with either Cy3 or Cy5 attached via a C_3_ linker to the 5′ end of the RNA. The structures have been solved at a resolution of 2.4 Å, from which the interaction between the dsRNA and the fluorophores could be observed. As expected, both Cy3 and Cy5 stack on the ends of the duplex RNA, but with an unexpected major difference in conformation from the corresponding DNA structures as deduced from NMR studies.

## Materials and Methods

### Sample preparation and purification

A gene encoding *T. thermophilus* L5 protein was synthesized. The gene expressing an N-terminal His_6_-MBP (maltose binding protein)-TEV (tobacco etch virus) cleavage site-L5 protein fusion was inserted into the pET28a vector (Novagen). After confirmation by sequencing, the plasmid containing the correct gene was transformed into fresh BL21(DE3) Rosetta competent cells (Invitrogen, Carlsbad, CA) for expression. A single colony was picked and incubated in 1 L Luria broth cell culture at 37°C for 8 h, and isopropyl β-D-1-thiogalactopyranoside added to a final concentration of 1 mM to induce protein expression at 37°C for 4 h. Cells were harvested and resuspended in 2× phosphate-buffered saline, 10 mM imidazole buffer, and frozen at −20°C. After thawing at room temperature, the cells were sonicated, and the lysate containing recombinant protein was purified using a Ni-nitrilotriacetic acid gravity column (GE Healthcare, Little Chalfont, UK). After elution using phosphate-buffered saline and 200 mM imidazole buffer, the protein was incubated with TEV protease to cleave the His_6_-MBP tag. The cleaved protein was then applied to heparin and size exclusion columns (GE Healthcare) for further purification. The purified L5 protein was concentrated to 0.1 mM and kept on ice.

### RNA synthesis

RNA was synthesized using UltraMILD ribonucleotidephosphoramidites with 2′O-*tert*-butyldimethyl-silyl protection (18, 19) (Link Technologies). Oligoribonucleotides were deprotected using anhydrous 2 M ammonia in methanol (Sigma-Aldrich, St. Louis, MO) for 36 h at room temperature, and evaporated to dryness. All oligoribonucleotides were redissolved in 115 *μ*L of anhydrous DMSO and 125 *μ*L triethylaminetrihyrofluoride (Sigma-Aldrich) to remove 2′O-*tert*-butyldimethyl-silyl groups, and agitated at 65°C in the dark for 2.5 h before butanol precipitation. RNA was purified by electrophoresis in 12% polyacrylamide under denaturing conditions in 90 mM Tris.borate (pH 8.3), 10 mM EDTA (Tris-borate EDTA buffer), and 7 M urea. RNA was electroeluted from excised gel slices using an Elutrap system (Whatman, Maidstone, UK) in 0.5 × Tris-borate EDTA buffer overnight at 4°C, recovered by ethanol precipitation, and redissolved in 5 mM HEPES (pH 7.0) and 20 mM NaCl.

### Crystallization

Purified RNA was annealed by slow cooling from 60°C, and its concentration was adjusted to 0.1 mM before mixing with an equal volume of L5 protein sample. The RNA-protein mixture was then gently mixed with 0.3 M Mg formate, 0.1 M bis-tris methane (pH 5.5), and 50 mM KF. This was sealed with 0.5 mL mother liquid at 4°C in hanging-drop mode and crystals of 20 × 20 × 200 *μ*m of space group P2_1_2_1_2 grew in 1 week. Crystals were flash frozen and stored in liquid nitrogen.

### Data collection and structure determination

The x-ray diffraction data sets were acquired at European Synchrotron Radiation Facility synchrotron beam lines. Initial phases were acquired by molecular replacement using PDB: 1MJI in the PHENIX suite (20). The structural model was manually adjusted and refined using Coot and PHENIX.refine. Crystallographic statistics are shown in Table 1. The Cy3 and Cy5 structures have been deposited with the PDB: 5NS4 and 5NS3, respectively.

**Table 1:** Details of Data Collection and Refinement Statistics for the Data as Deposited in the PDB

	Cy3-U	Cy5-C
**Data Collection**		

Wavelength (Å)	1.00	1.00
Resolution range (Å)	46.88–2.4 (2.486–2.4)	45.08–2.4 (2.486–2.4)
Space group	P2_1_2_1_2	P2_1_2_1_2
Unit cell	93.76 122.43 51.01 Å	95.04 123.90 51.20 Å
	90, 90, 90°	90, 90, 90°
Unique reflections	23654 (2309)	24351 (2367)
Completeness (%)	100 (100)	100 (100)
Wilson B-factor	48.88	48.86

**Refinement**		

R-work	0.2586 (0.3159)	0.2525 (0.3308)
R-free	0.2817 (0.3374)	0.2727 (0.3577)

**Number of Atoms**		

Macromolecules	3589	3594
Ligands	40	50

**RMSD**		

Bond lengths (Å)	0.013	0.014
Bond angles (**°)**	1.53	1.56

**B-Factors**		

Average B-factor	37.09	35.33
Macromolecules	36.56	34.73
Ligands (Cy3/5)	77.99	75.03

**PDB**		

PDB	5NS4	5NS3

Statistics for the highest resolution shell are in parentheses. RMSD, root mean square deviation.

## Results

### Crystallization of cyanine fluorophore-conjugated RNA complexed with L5 protein

A synthetic gene encoding *T. thermophilus* L5 was cloned into vector pET28a (Novagen) to create a gene expressing (from the N- to C-termini) His_6_-MBP gene-TEV cleavage site-L5 protein. The fusion was expressed in in *Escherichia coli* BL21(DE3) and purified by affinity chromatography on Ni-nitrilotriacetic acid. After cleavage of the fusion with TEV protease, the free L5 protein was purified by ion exchange and gel filtration chromatography.

Thirty-three nucleotide RNA species designed to form flush-ended stem-loop structures (Fig. 1) were chemically synthesized in four forms, with each of the possible Watson-Crick basepairs closing the free end of the hairpin. Cy3 and Cy5 fluorophores were coupled to each as a phosphoramidite as the final step of the synthesis. This results in fluorophore coupled to the 5′ phosphate group via a C_3_ methylene linker.

Purified L5 protein was mixed in equimolar proportions with either Cy3- or Cy5-conjugated RNA and set up for crystallization trials. The protein-RNA complexes that ultimately yielded the best diffracting crystals had terminal 5′U-3′A and 5′C-3′G for Cy3 and Cy5, respectively. Crystals for both constructs grew up to 0.2 mm at the longest dimension in a week. Both grew in space group P2_1_2_1_2 with closely similar cell dimensions and diffracted to a resolution of 2.4 Å (Table 1). Initial phases were acquired by molecular replacement using the structure of *T. thermophilus* L5 PDB: 1MJI (17) and the structures were subsequently refined.

### The structure of the RNA-protein complexes

The asymmetric unit in both crystal structures contain two complete complexes of an RNA stem-loop bound to the L5 protein. The protein structure is identical to that determined by Perederina et al. (17) (root mean square deviation [RMSD] 0.394 Å), despite being determined in different spacegroups, with an *α*1-*β*1-*α*2-*β*2-*β*3-*α*3-*β*4-*β*5-*α*4 secondary structure (Fig. S1). One face of the five-strand antiparallel *β*-sheet is largely covered by the four *α*-helices, whereas the other is open.

For each structure, the two RNA molecules adopt the same conformation (RMSD 0.357 Å) and are related by a noncrystallographic two-fold axis between the two terminal loops. The conformation of the RNA is closely similar to the structure of Perederina et al. (17), with overall RMSD values of 0.426 Å. Both RNA structures are essentially A-form helical, with a single helical axis (Fig. S2). The segment between C35 and U48 forms a series of noncanonical base interactions in a helical conformation, including triple interactions such as C38-G44-C47, above which lies the terminal loop. The loop makes a sharp turn at G41, followed by C42, C43, and A45, which are stacked on one face of the loop. U40 and A45 are coplanar and stacked upon the A39-A46 basepair. The open end of the helix forms nine Watson-Crick basepairs, with a two-nucleotide bulge that leaves five uninterrupted Watson-Crick basepairs adopting a standard A-form helix at the terminus. The cyanine fluorophores are stacked upon the terminal base basepair as discussed below.

**Figure 2:**
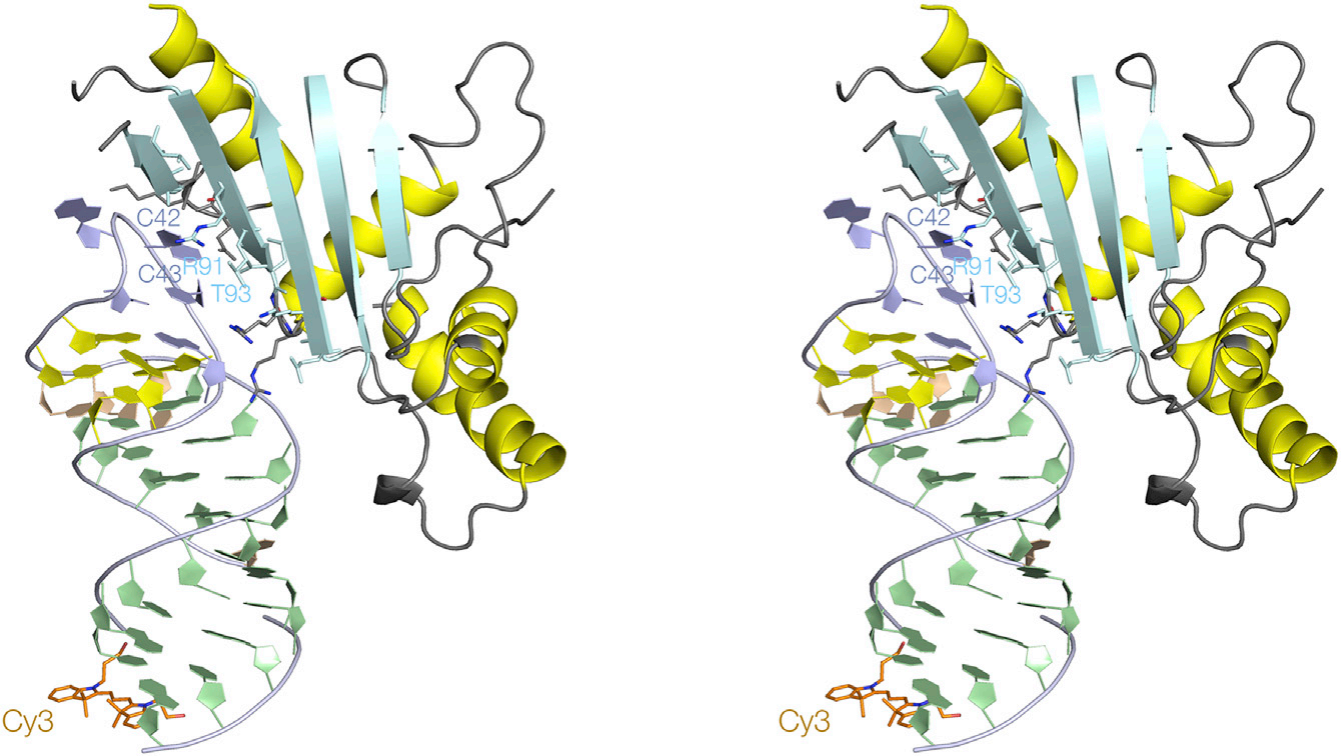
The crystal structure of one RNA-protein complex with Cy3 attached to the 5′-terminus of the RNA. A parallel-eye stereoscopic view of the structure is shown, with the L5 protein depicted in cartoon mode with *α*-helices (*yellow*) and *β*-sheet (*cyan*). To see this figure in color, go online.

In each complex, the protein interacts with the minor groove face of the terminal loop (Fig. 2). The stacked nucleotides C42 and 43 make contact with *β*3 (88–94) and basic side chains extend from the extreme N-terminal end of *α*3 (R95 and R96) toward the backbone, together with K67 from the *α*2-*β*2 linking section. The distal end of the RNA is far removed from the directly bound L5 protein.

The asymmetric unit comprises two RNA-protein complexes in which the RNA stem-loops are associated at the loop end (Fig. S3), with a hydrogen bond formed between the O2' atoms of the two A46 ribose groups. In addition, C43 N4 is hydrogen bonded to the *R*_p_ nonbridging phosphate oxygen atom of C47 of the other RNA. The axes of the two RNA molecules are inclined with an included angle of 120°. Within the dimer, the minor groove of the open end of the RNA helix is juxtaposed with the open planar face of the *β*-sheet of the protein that is bound to the loop of the other RNA in the dimer. However, we observe no protein electron density at the end of the helix, at the position where the fluorophores are located.

### Interactions in the crystal lattice: two different environments for the fluorophores

Although the relative disposition of RNA and protein within the asymmetric unit could not result in interference with the position of the fluorophore, it is important to examine contacts between the dimer with other neighboring molecules within the lattice. This system was chosen because of the unobscured environment of the open end of the RNA helix. However, our constructs crystallized in a different space group (P2_1_2_1_2) from the original published structure (that was P2_1_) (17), so crystal contacts will not be the same.

**Figure 3:**
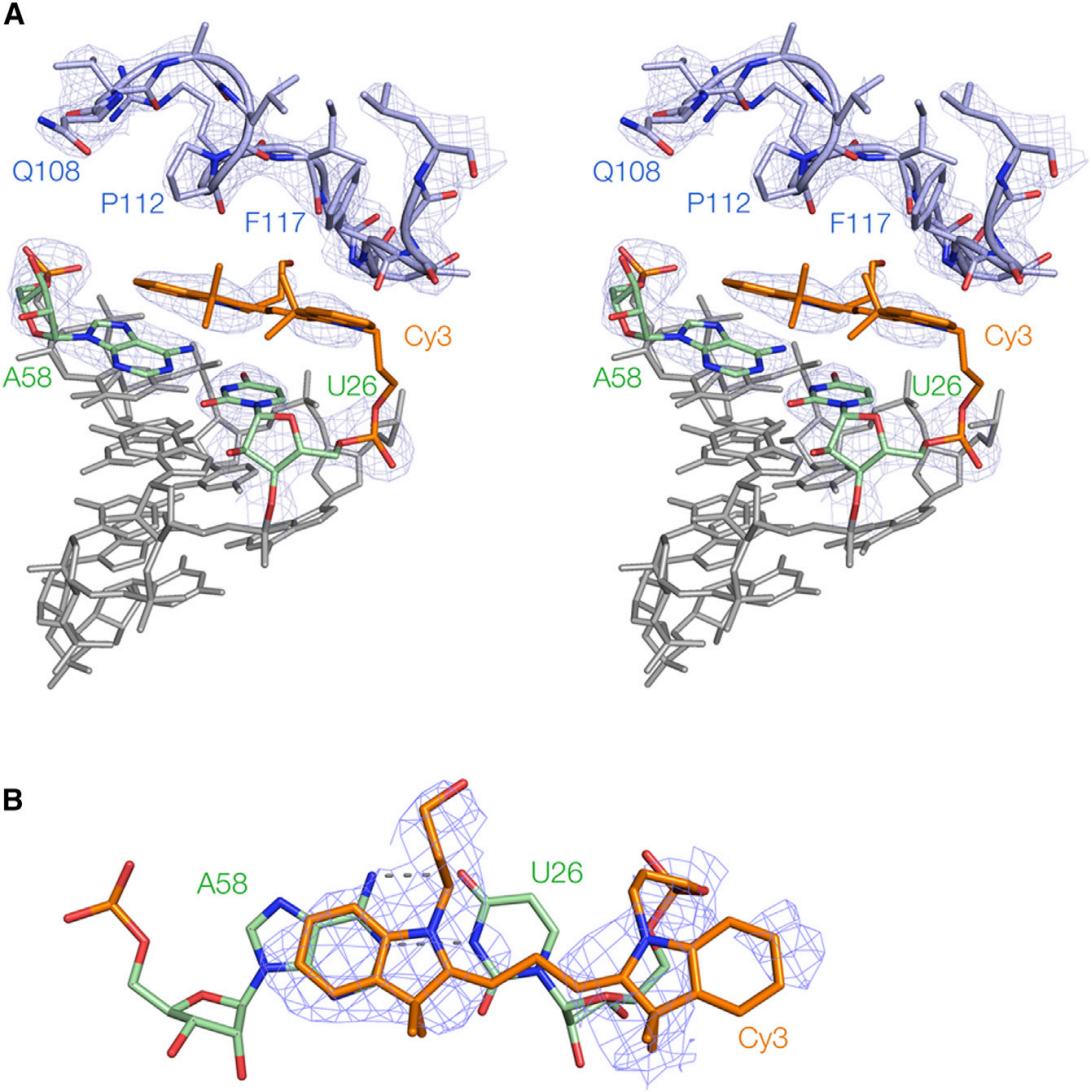
Cy3 at the RNA-protein interface. Cy3 is colored orange, and the terminal basepair of the RNA (U26-A58) colored green. The arch of the adjacent L5 protein molecule comprising amino acids 106–120 shown in blue encloses a cavity in which the fluorophore is located. The ***F***_O_-***F***_C_ electron density omit map is shown for the key elements of the structure. (*A*) A parallel-eye stereoscopic side view of Cy3 stacked on the end of the RNA in the complex, showing the arch of the protein. (*B*) Top view of the Cy3 stacked on the U26-A58 basepair. To see this figure in color, go online.

Because the asymmetric unit is a dimer, the two open ends where the fluorophores are attached are not related by crystal symmetry, and their local environments are not equivalent. This results in very different environments for the two open ends of the RNA in each dimer. Within the crystal lattice dimeric complexes are lined up along their long axes (Fig. S4). For any dimer, the open end (with the attached fluorophore) of one RNA is almost coaxial with that of the adjacent dimeric complex, and these RNA backbones are within 8 Å between the two dimers. By contrast, the corresponding end of the other RNA within the dimer is > 20 Å from the RNA of the adjacent dimer, but in this case the arginine-rich loop between *α*3 and *β*4 (residues 113–120) forms an arch over the end of the RNA, leaving a cavity in which the fluorophore is located (Figs. 3 *A* and S5). These different environments have contrasting consequences for the attached fluorophore. At the end with the protein arch we observe electron density for both Cy3 and Cy5, whereas at the end with the RNA-RNA interaction no fluorophore density was observed for Cy3, and weak density for Cy5. In this latter case, it is likely that the fluorophore is very mobile.

### The conformation of the Cy5 fluorophore on the RNA

**Figure 4:**
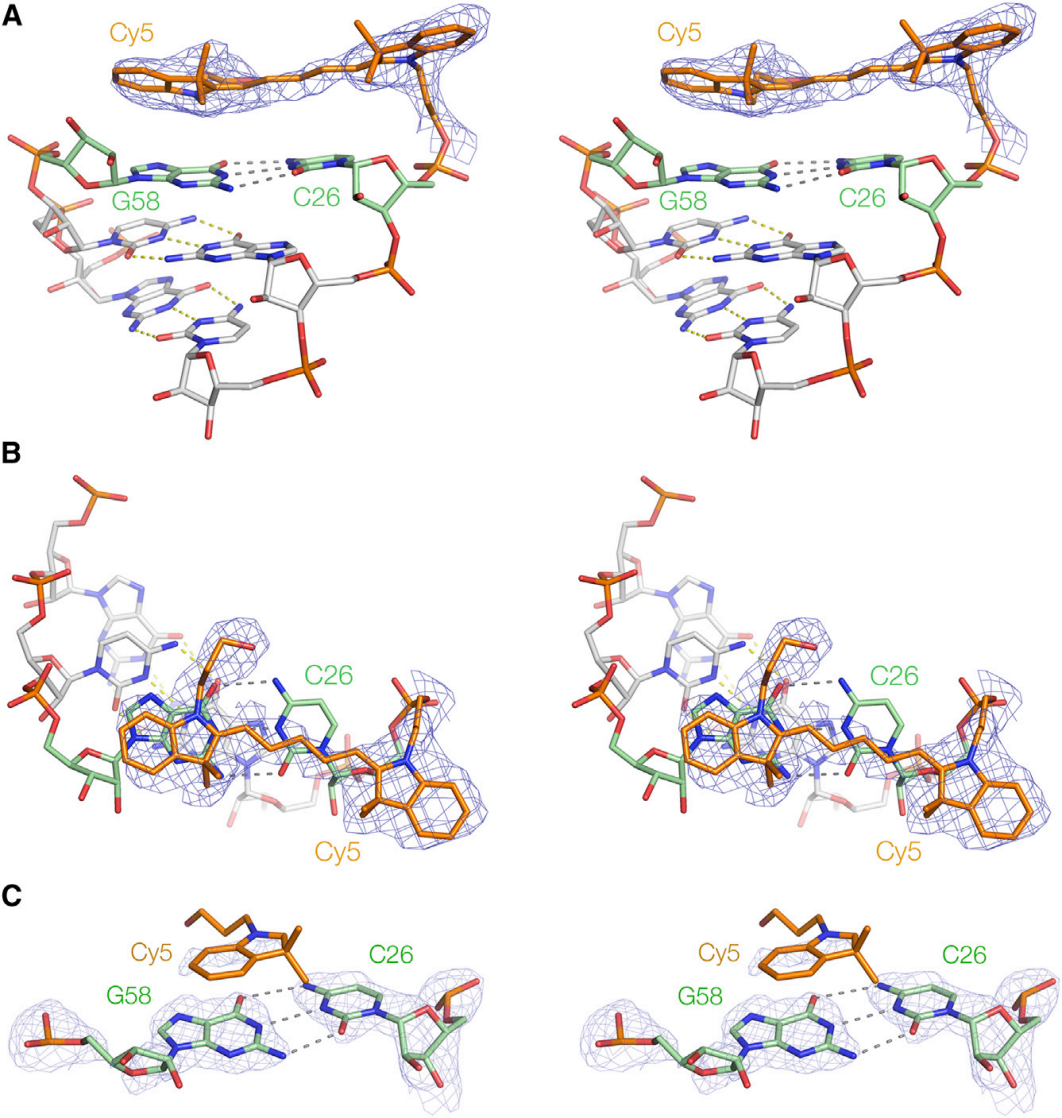
The structure of Cy5 attached to the RNA. Cy5 is colored orange, and the terminal basepair of the RNA (C26-G58) colored green. The ***F***_O_-***F***_C_ electron density omit map is shown for the fluorophore, and for the terminal basepair (in (*C*) only). Parallel-eye stereoscopic views are shown. (*A* and *B*) Cy5 stacked on the C26-G58 basepair at the RNA-protein end of the RNA in the complex. Side view (*A*) and top view (*B*). (*C*) Top view looking down onto the Cy5 stacked onto the terminal basepair at the RNA-RNA interface. Electron density is only visible for the distal indole ring stacked on G58. To see this figure in color, go online.

The electron density for Cy5 attached at the RNA terminus at the RNA-protein interface (Fig. 4, *A* and *B*) is well defined for the whole fluorophore, including the C_5_ polymethine section. The electron density map shows that Cy5 is stacked upon the terminal basepair, with both rings of the distal indole stacked over the guanine nucleobase (G58) (average spacing 3.9 Å). The C_5_ polymethine section sits over the cytosine nucleobase (C26) and the proximal indole is directed out into the solvent. The long axis of the fluorophore is rotated 10° about the helical axis relative to that of the terminal C-G basepair. Both the polymethylene linkers are well defined in the omit map, showing that the Cy5 fluorophore is stacked on the face that places its indole nitrogen atoms on the major groove side of the C-G basepair. Electron density for the Cy5 at the RNA-RNA interface is much more poorly defined (Fig. 4 *C*), although there is some density indicating the position of the indole ring stacked over G58. Average B-factors for this Cy5 were high (Table 2). Evidently, this fluorophore is more mobile, yet is partially localized stacked on the end of the helical RNA.

**Table 2:** Average B-Factors for Cy3 and Cy5 Fluorophores and Their Adjacent Terminal Basepairs

Fluorophore or Basepair	Crystal Interface	Average B-Factor
Cy3	RNA-protein	78.0
A58-U26	RNA-protein	45.4
Cy5	RNA-protein	64.5
G58-C26	RNA-protein	32.1
Cy5	RNA-RNA	99.6
G58-C26	RNA-RNA	48.7

Debye-Waller B-factors were calculated as B = 8*π*^2^ <u^2^>, where u is the mean atomic displacement.

### The conformation of the Cy3 fluorophore on the RNA

In general, the electron density for Cy3 was weaker than that observed for the Cy5 fluorophore, and average B-factors were higher (Table 2). No electron density was observable for Cy3 at the RNA-RNA interface, but we did see clear density at the RNA-protein interface (Fig. 3). This was weaker than that for the RNA helix, consistent with our previously deduced lateral mobility of terminally attached fluorophores (10). An omit map of the fluorophore shows that the two indole rings are clearly defined, including the polymethylene chain extending from the distal indole nitrogen atom. If the *σ* level is lowered then the C_3_ linker connecting the proximal indole ring to the 5′ phosphate of the RNA becomes clearly visible, but no density for the C_3_ polymethine section linking the two indole rings can be observed. The distal indole ring is stacked on the purine ring of the terminal adenine (A58), with an excellent overlap between the six-membered rings at a mean distance of 3.4 Å. By contrast, the five-membered ring of the proximal indole lies above the ribose of U26, with the six-membered ring directed out into the solvent, and the C_3_ polymethine section lies above its pyrimidine nucleobase. The centers of the regions of density corresponding to the two indole ring systems shows that the long axis of the Cy3 is close to parallel with that of the terminal U-A basepair. In addition, the density for the polymethylene chain unambiguously demonstrates that the indole nitrogen atoms of the fluorophore are located on the major groove side of the terminal U-A basepair.

## Discussion

Here we present, to our knowledge, the first x-ray crystallographic structural analysis of cyanine fluorophores attached to dsRNA tethered through the terminal 5′ phosphate. This was carried out using a *T. thermophilus* L5 protein-RNA complex that creates two different environments for the free end of the dsRNA. This system could be of general utility for the study of molecules interacting with the end of RNA species.

Our crystallographic studies confirm the predisposition of Cy3 and Cy5 to stack on the final basepair of double-stranded nucleic acids. We have noted that this is true irrespective of the identity of the sequence of the terminal basepair, although we present only the best-defined structures for each fluorophore here, i.e., Cy3 on U-A and Cy5 on C-G. Terminal stacking is consistent with earlier data measuring FRET efficiency between Cy3 and Cy5 tethered to the two 5′-termini of double stranded DNA and RNA helices (10, 11). In those experiments, *E*_FRET_ exhibited a sinusoidal variation with helix length consistent with terminal stacking of both fluorophores.

However, those FRET experiments were not consistent with a fixed orientation of the cyanine fluorophores on the end of the RNA, but rather the width of the modulation of the dependence of *E*_FRET_ on helix length indicated a lateral motion of significant amplitude (10, 11). Moreover, multiple fluorescent lifetimes of the tethered fluorophores indicated that a fraction of the population is unstacked at a given moment (10, 13, 14). Thus, although the FRET results suggested a strong tendency of the cyanine fluorophores to adopt a terminally-stacked conformation, they were clearly subject to significant flexible motion in terms of both lateral and unstacking movements (10, 11). The crystal structures are also indicative of local motion by the fluorophores. The electron density of both fluorophores (but particularly Cy3) is less well defined than that for the RNA, and the average B-factors are higher than the adjacent terminal basepair in each case (Table 2). At the RNA-protein interface, the loop of the opposing protein will probably constrain the unstacking dynamics, helping us observe the stacked fluorophores. Yet some density is observed for Cy5 at the less-constrained RNA-RNA interface, so that fluorophore stacking is not solely a product of the constraint observed at the other interface. Terminal stacking is intrinsic to the cyanine fluorophores attached to double-stranded RNA, consistent with the earlier FRET experiments (10, 11). We note that there is some correlation between the average B-factors of the fluorophores (in the order Cy5 (RNA-protein interface) < Cy3 (RNA-protein interface) < Cy5 (RNA-RNA interface)) (Table 2) with those of the adjacent basepairs, indicating that the fluorophore stabilizes the basepair on which it is stacked.

**Figure 5:**
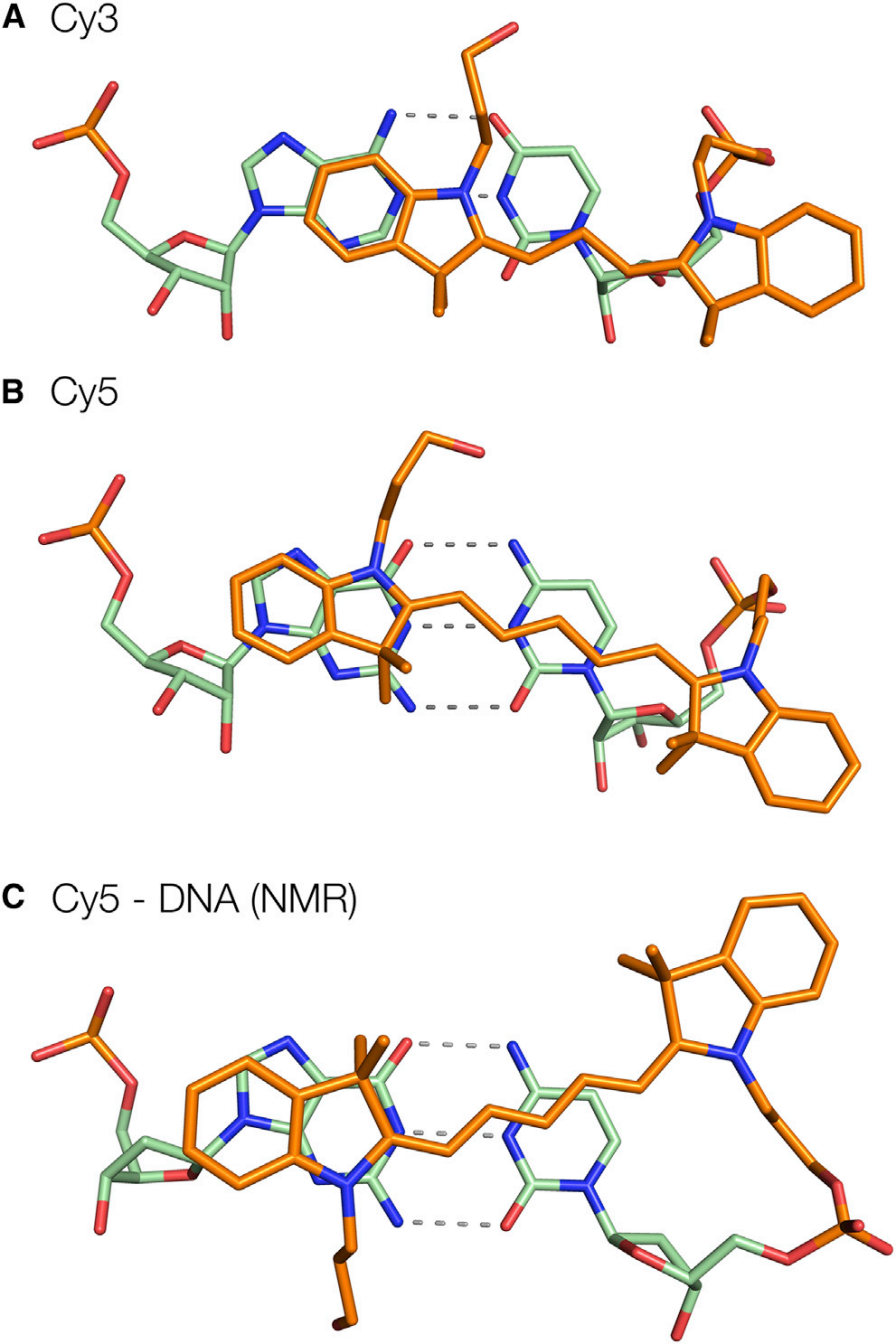
The locations of Cy3 (*A*) and Cy5 (*B*) observed stacked on the terminal basepair of the RNA helix in the crystal, compared with Cy5 stacked on the terminal basepair of a DNA helix observed previously by NMR (*C*) (10). To see this figure in color, go online.

The conclusions from the crystallography are generally consistent with the previous results using FRET (10, 11). However, a comparison of the structures of the fluorophores stacked on an RNA helix with those from earlier NMR studies of fluorophores attached to DNA duplexes reveals a completely unexpected result (Fig. 5). The crystallographic data show that both Cy3 and Cy5 (Fig. 5, *A* and *B*, respectively) stack on RNA such that the indole nitrogen atoms lie on the major groove side of the terminal basepair, placing the pendant methyl groups on the minor groove side. This is well defined by the data, because the electron density of the polymethylene chains is extremely clear in both structures (Figs. 3 *B* and 4 *B*). By contrast, in the DNA structures (exemplified by Fig. 5 *C*), the fluorophore is rotated 180° about its long axis, so that the indole nitrogen atoms are now on the minor groove side of the terminal basepair (9, 10). An NMR structure of sulfoindocarbocyanine-3 attached to the terminal phosphate of DNA via a longer linker indicated the same rotational setting about the fluorophore long axis (12). The orientation about the long axis is not defined by the FRET data, and this difference between DNA and RNA was not anticipated. For both DNA and RNA, the distal indole ring is well stacked over the A or G nucleobase, but the differing rotational setting alters the position of the fluorophores overall, so that the proximal indole ring in RNA locates over the 5′-terminal ribose, whereas in DNA it was placed on the major groove side of the basepair.

We have asked the question whether this difference between the conformations of the cyanine fluorophores on dsRNA and DNA is correct, or perhaps a result of the environment in the crystal forcing the fluorophores to adopt a different conformation. Although the fluorophores at the RNA-protein interface make no direct contact with the protein, this might not be the case for the alternative rotational setting. To examine this possibility, we used molecular modeling to replace the Cy5 in the crystal environment with that taken from the NMR structure attached to dsDNA, i.e., having the rotational setting about its long axis that places the indole nitrogen atoms on the minor groove side of the terminal basepair. In the resulting model, we find that the fluorophore makes no steric clash with the protein (Fig. S6 a). Although there could be some conformational influence from the environment in the crystal lattice, we can exclude the possibility that the DNA-like conformation of the fluorophores is excluded by a significant steric clash. In addition, the model suggests an origin for the difference between the conformation of the cyanine fluorophores attached via a short tether to dsDNA and RNA. The altered trajectory of the tether resulting from the C_3′_-*endo* pucker of the terminal ribose will tend to direct the fluorophore into a different position, imparting a large rotation of the fluorophore about the helical axis (Fig. S6 b), which is not consistent with experimental FRET data. This suggests that the observed conformational difference between the cyanine fluorophores attached to dsRNA and dsDNA is real, resulting from the different structure of the two polymers.

The conformation we observe in the crystal is consistent with experimental FRET data. Furthermore, we used the crystal structures to create models of dsRNA with terminally attached Cy3 and Cy5 exactly as observed in the crystal, generating a series of duplexes with lengths from 10 to 22 bp. We calculated *E*_FRET_ from the geometry of each (using standard geometric and spectroscopic parameters, and allowing the fluorophores to undergo lateral motion), finding the expected modulation with helix length (Fig. S7). These provide reasonable agreement with experimental data obtained on a DNA-RNA hybrid duplex using the same three-carbon tether (10).

The average position of the cyanine fluorophores terminally attached to dsRNA in the crystal structures is unambiguous. This information will be important in the interpretation of distance and angular information in FRET experiments with RNA species. When the position and orientation of the fluorophores are known, then potentially both angular and distance information within the attached macromolecule can be deduced.

## Conclusions

X-ray crystallography shows the strong tendency of the indodicarbocyanine fluorophores Cy3 and Cy5 terminally attached to double-stranded RNA to stack onto the ends of the molecule. The fluorophores are stacked on the terminal basepair such that their indole nitrogen atoms lie on the major groove side.

## Author Contributions

Y.L. performed experimental work and crystallographic analysis. Y.L. and D.M.J.L. planned experiments and analyzed data. D.M.J.L. wrote the article.

## Supporting Material

Document S1. Figs. S1–S7

Document S2. Article plus Supporting Material

## References

[1] Lilley D.M., Wilson T.J. (2000). Fluorescence resonance energy transfer as a structural tool for nucleic acids. Curr. Opin. Chem. Biol..

[2] Clegg R.M. (2002). FRET tells us about proximities, distances, orientations and dynamic properties. J. Biotechnol..

[3] Ha T. (2004). Structural dynamics and processing of nucleic acids revealed by single-molecule spectroscopy. Biochemistry.

[4] Haas E., Katchalski-Katzir E., Steinberg I.Z. (1978). Effect of the orientation of donor and acceptor on the probability of energy transfer involving electronic transitions of mixed polarization. Biochemistry.

[5] Dale R.E., Eisinger J., Blumberg W.E. (1979). The orientational freedom of molecular probes. The orientation factor in intramolecular energy transfer. Biophys. J..

[6] Wu P., Brand L. (1992). Orientation factor in steady-state and time-resolved resonance energy transfer measurements. Biochemistry.

[7] Mujumdar R.B., Ernst L.A., Waggoner A.S. (1993). Cyanine dye labeling reagents: sulfoindocyanine succinimidyl esters. Bioconjug. Chem..

[8] Ernst L.A., Gupta R.K., Waggoner A.S. (1989). Cyanine dye labeling reagents for sulfhydryl groups. Cytometry.

[9] Norman D.G., Grainger R.J., Lilley D.M.J. (2000). Location of cyanine-3 on double-stranded DNA: importance for fluorescence resonance energy transfer studies. Biochemistry.

[10] Iqbal A., Arslan S., Lilley D.M.J. (2008). Orientation dependence in fluorescent energy transfer between Cy3 and Cy5 terminally attached to double-stranded nucleic acids. Proc. Natl. Acad. Sci. USA.

[11] Ouellet J., Schorr S., Lilley D.M. (2011). Orientation of cyanine fluorophores terminally attached to DNA via long, flexible tethers. Biophys. J..

[12] Urnavicius L., McPhee S.A., Norman D.G. (2012). The structure of sulfoindocarbocyanine 3 terminally attached to dsDNA via a long, flexible tether. Biophys. J..

[13] Sanborn M.E., Connolly B.K., Levitus M. (2007). Fluorescence properties and photophysics of the sulfoindocyanine Cy3 linked covalently to DNA. J. Phys. Chem. B.

[14] Spiriti J., Binder J.K., van der Vaart A. (2011). Cy3-DNA stacking interactions strongly depend on the identity of the terminal basepair. Biophys. J..

[15] Ouellet J., Melcher S., Lilley D.M. (2010). Structure of the three-way helical junction of the hepatitis C virus IRES element. RNA.

[16] Fessl T., Lilley D.M.J. (2013). Measurement of the change in twist at a helical junction in RNA using the orientation dependence of FRET. Biophys. J..

[17] Perederina A., Nevskaya N., Nikonov S. (2002). Detailed analysis of RNA-protein interactions within the bacterial ribosomal protein L5/5S rRNA complex. RNA.

[18] Hakimelahi G.H., Proba Z.A., Ogilvie K.K. (1981). High yield selective 3ʹ-silylation of ribonucleosides. Tetrahedron Lett..

[19] Perreault J.-P., Wu T.F., Cedergren R. (1990). Mixed deoxyribo- and ribo-oligonucleotides with catalytic activity. Nature.

[20] Adams P.D., Afonine P.V., Zwart P.H. (2010). PHENIX: a comprehensive Python-based system for macromolecular structure solution. Acta Crystallogr. D Biol. Crystallogr..

