# Supplementary material for: Crystal Structures of Cyanine Fluorophores Stacked onto the End of Double-Stranded RNA
Source: Biophys J. 2017 Dec 5;113(11):2336–43. doi: 10.1016/j.bpj.2017.10.002 (PMC5768521; doi:10.1016/j.bpj.2017.10.002)
Supplement: Document S1. Figs. S1–S7 [file mmc1.pdf]

Biophysical Journal, Volume *113*

Supplemental Information

Crystal Structures of Cyanine Fluorophores Stacked onto the End of Double-Stranded RNA

Yijin Liu and David M.J. Lilley

SUPPLEMENTARY FIGURES

A

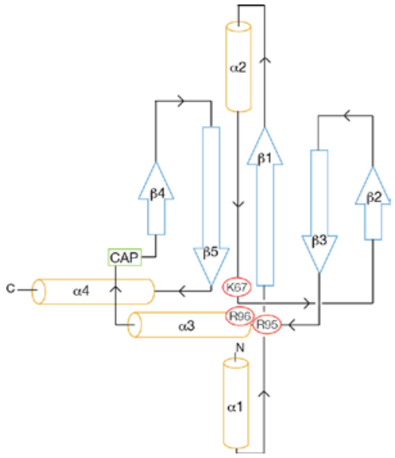

B

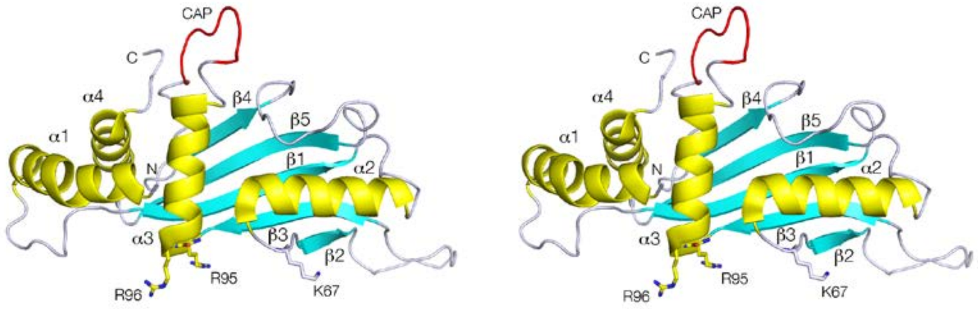

C

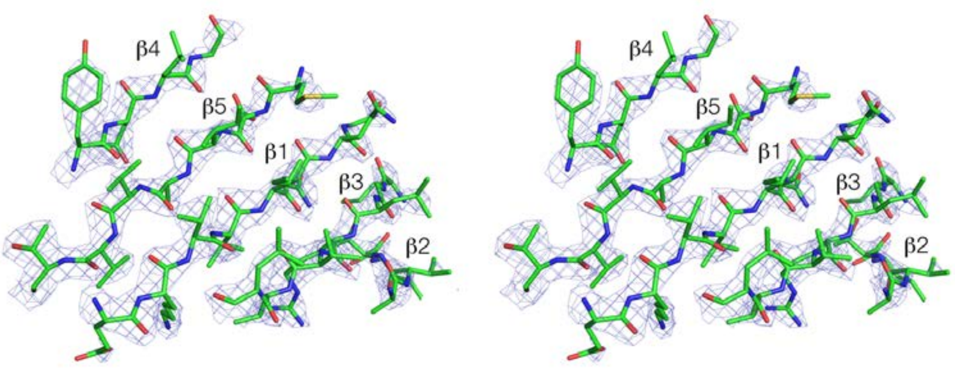

Figure S1. Structure of L5 protein in the complex with RNA-Cy3.

A. Schematic to show the topological connection between regions of α-helix (yellow) and β-sheet (cyan) in the secondary structure.

B. Parallel-eye stereoscopic view of the crystal structure of one protein monomer. The region that caps the fluorophore in an adjacent complex is colored red (CAP), and amino acid side chains interacting directly with the RNA backbone are indicated in stick form.

C. Representative electron density. $2\mathbf{F}_o$-$\mathbf{F}_c$ map contoured at 2σ for the antiparallel β-sheet.

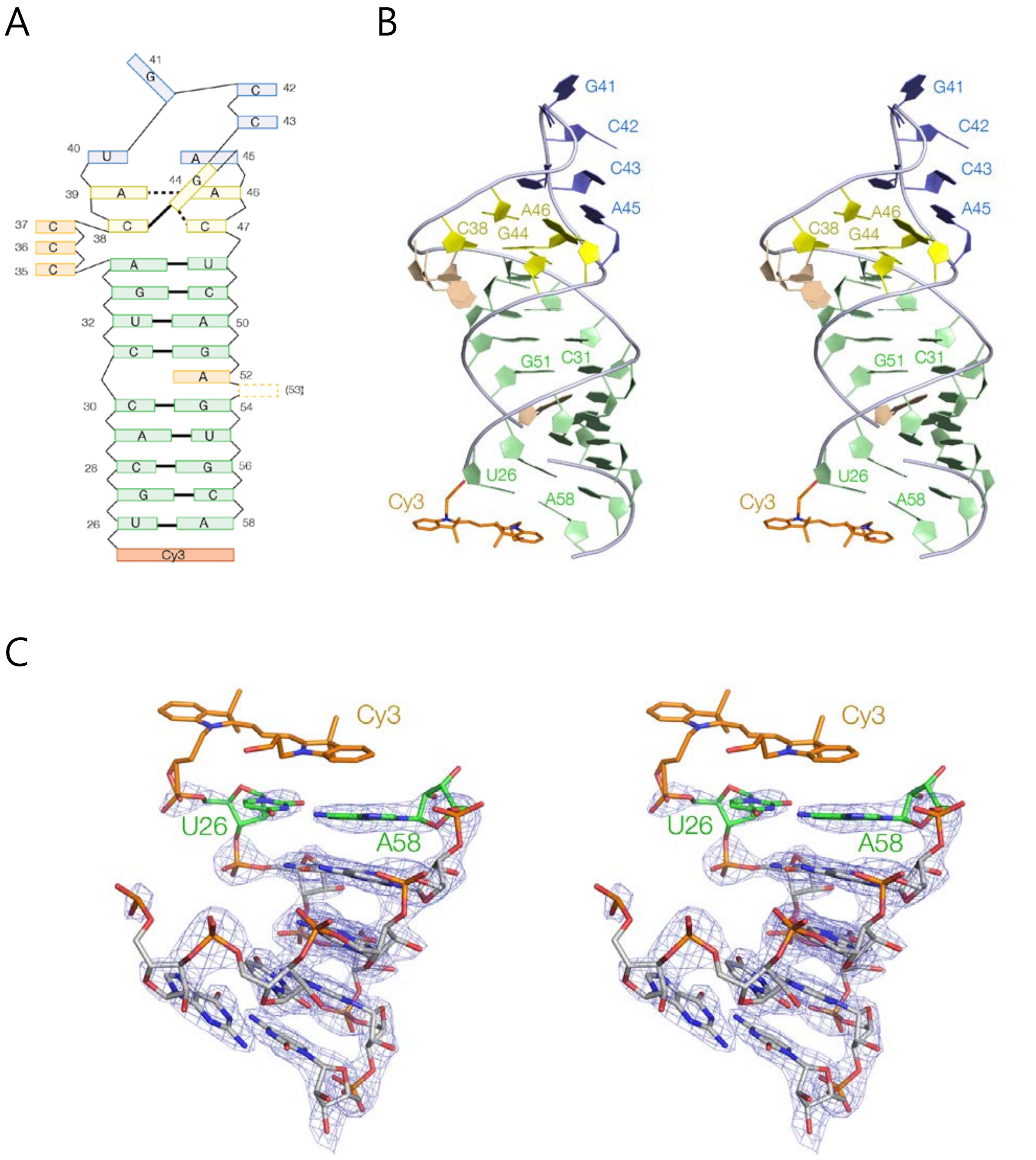

Figure S2. Structure of the RNA in the complex with RNA-Cy3.

A. A scheme showing the structure of the RNA in the complex with the L5 protein.

B. The crystal structure of the RNA in the complex with RNA-Cy3.

C. Representative electron density of the RNA at the RNA-protein interface. $2\mathbf{F}_o$-$\mathbf{F}_c$ map contoured at 2σ for the five basepairs adjacent to the Cy3 fluorophore.

B and **C** both are shown as parallel-eye stereoscopic views.

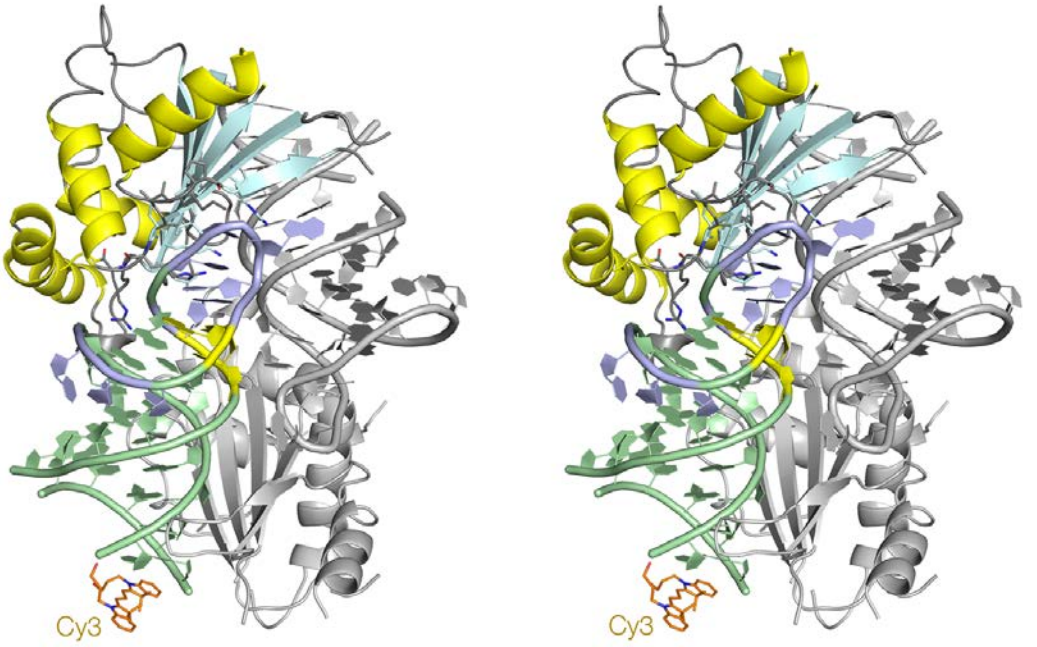

Figure S3. A parallel-eye stereoscopic view of the asymmetric unit of the crystal, comprising a dimer of the RNA-protein complex. One is colored while the other is shown in grey.

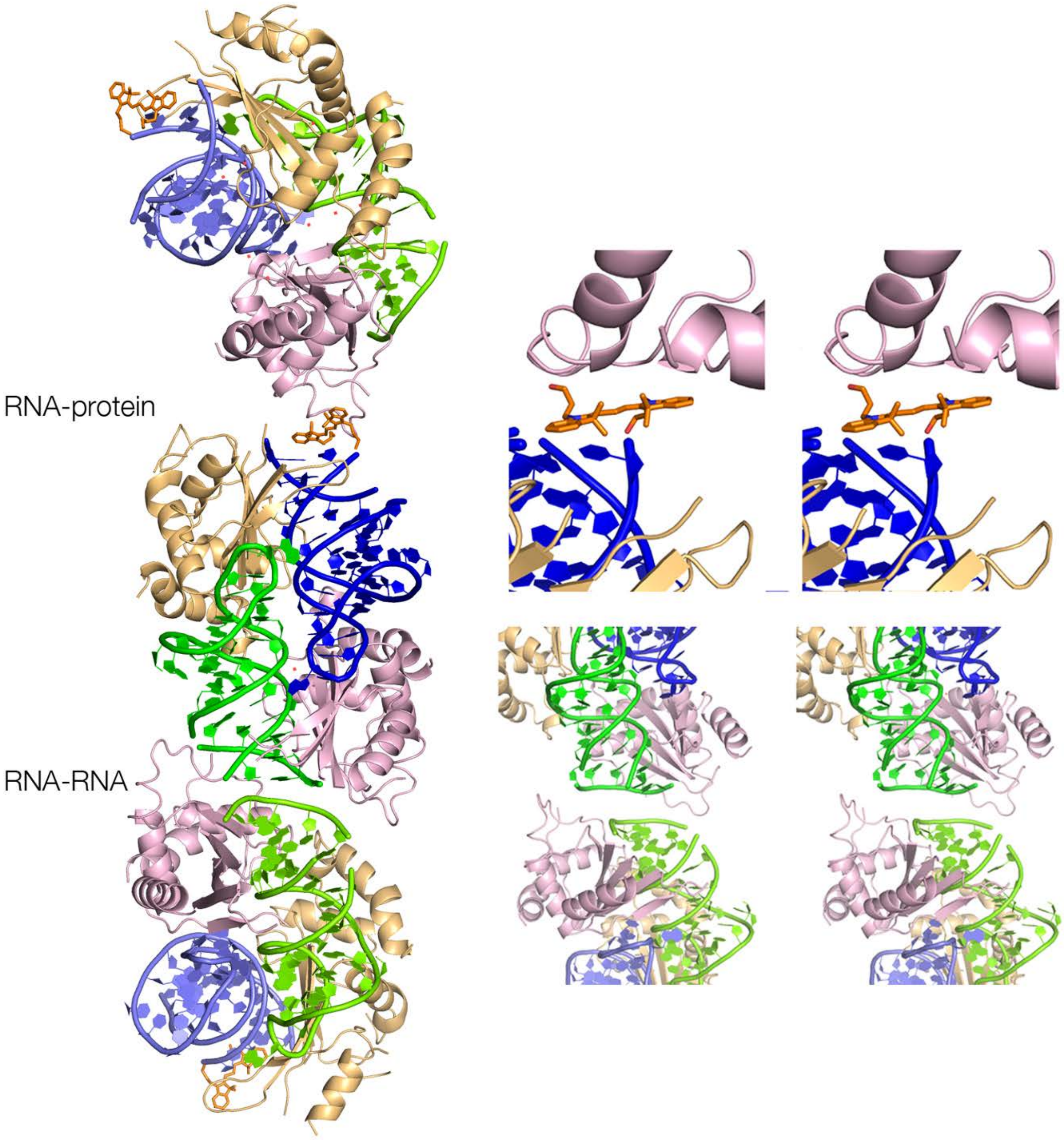

Figure S4. The association of asymmetric units within the crystal lattice. Three dimeric complexes are shown, with their interfaces expanded and shown as parallel-eye stereoscopic pairs. The upper one is the RNA-protein interface, where the Cy3 fluorophore is well defined. The lower one is the RNA-RNA interface.

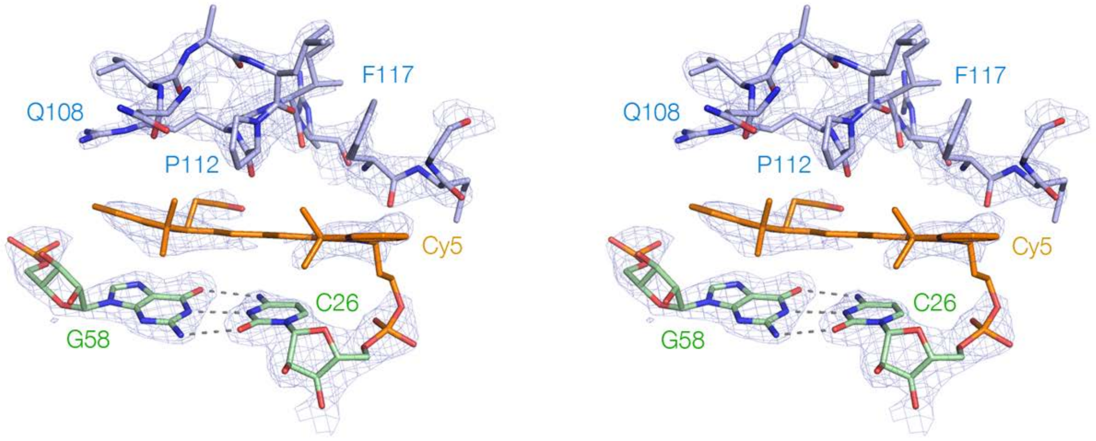

Figure S5. Parallel-eye stereoscopic view of the structure of Cy5 in the cavity between successive dimeric complexes at the protein-RNA interface. The 2$\mathbf{F}_o$-$\mathbf{F}_c$ electron density map contoured at 1.5 σ is shown for the polypeptide section between N108 and L120, the C-G terminal basepair of the RNA and the Cy5 fluorophore. This is essentially the same as the environment of Cy3 in the corresponding crystal shown in Figure 3A.

A

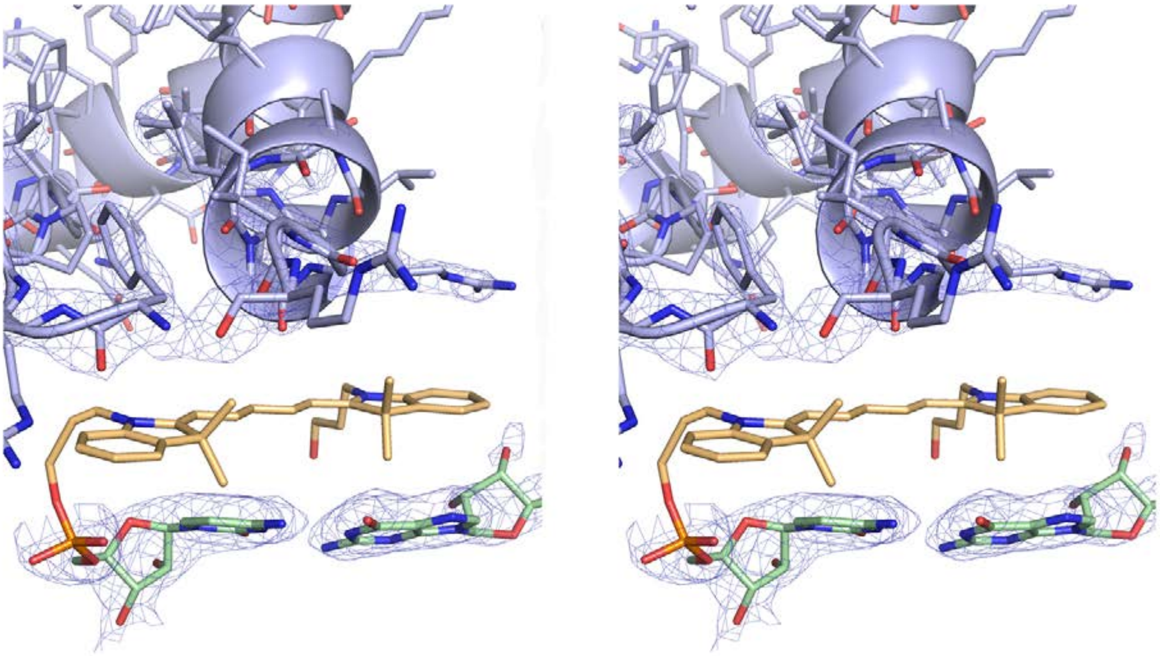

B

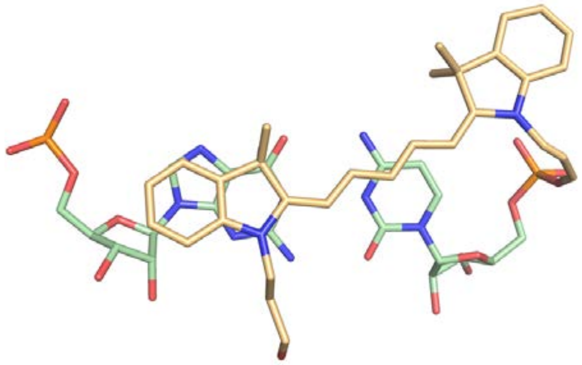

Figure S6. Accommodation of Cy5 in the conformation previously observed for dsDNA by NMR (with indole N atoms on the minor groove side) modelled into the crystal structure with dsRNA.

A. Parallel-eye stereoscopic view of the RNA-protein end showing the position of the modelled Cy5. Note that there is no significant steric clash with the protein.

B. View of the modelled Cy5 stacked on the terminal base pair. Comparison with the DNA structure shown in Figure 5C shows that the ribose conformation in the RNA directs the trajectory of the linker differently from DNA.

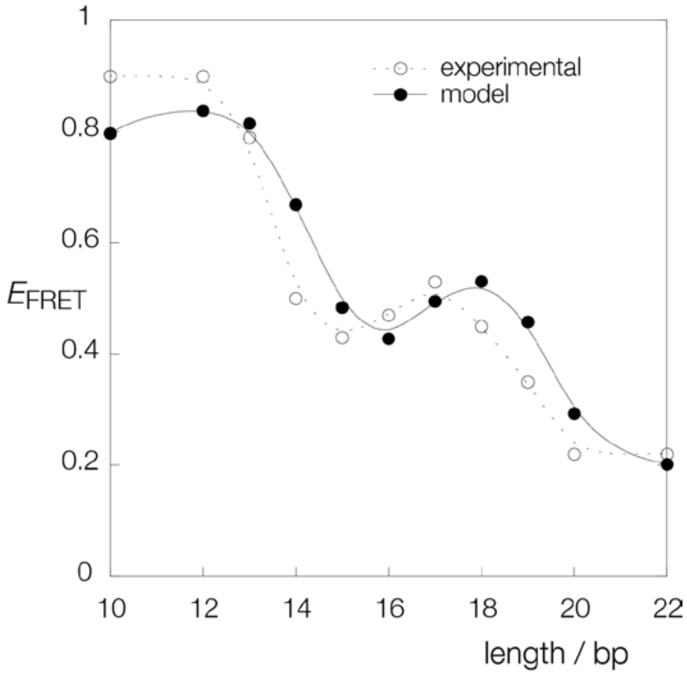

Figure S7. Calculated dependence of FRET efficiency for a series of terminally- Cy3- and Cy5-labelled duplex species from 10 - 22 bp. Using the structures of the cyanine fluorophores from the crystal structures, a series of RNA duplex models were generated using standard A-form geometry. The angles of the projected transition dipoles were allowed to vary laterally in a Gaussian dependence with a half-width of 50°, and the mean κ^2 calculated and used to calculate E_{FRET} for each species using values of refractive index n=1.33, donor quantum yield Q=0.35 and overlap integral J=5.4e-13 established previously. The mean E_{FRET} values are plotted against helix length (filled circles). The line is a just a smooth fit to the data to make the series clearer. The open circles are data for a duplex series of DNA:RNA hybrids using the same fluorophore species and three-carbon linkers used in the crystal structures. There is good agreement between the calculated structures and the experimental data. There is a small phase shift between the two sets of points, likely due to structural differences between DNA:RNA and dsRNA, and the differences in the terminal sequences. The original analysis and data were presented in Iqbal *et al.* 2008, Orientation dependence in fluorescent energy transfer between Cy3 and Cy5 terminally-attached to double-stranded nucleic acids. Proc. Natl. Acad. Sci. USA 105:11176-11181.
